# Can Animals Talk?

**Published:** 1880-12

**Authors:** 


### CAN ANIMALS TALK.

### INTERESTING FACTS IN REGARD TO BIRD LANGUAGE.

In the course of a very able paper on this subject the Bishop of Carlisle says :—"A dog sometimes looks as though he was thinking a thing out, and dog stories are very wonderful; but after all, the cleverest dog that ever lived yet has never been able to get beyond 'Bow-wow,' and we may safely predict that no dog will ever acquire even the simplest elements of human knowledge.

"But what, let us ask, is the real barrier between the dog's mind (if the term may be used) and the simplest elements of human knowledge? It consists in this fact; that the vocal organs of the dog are so constructed that it is impossible for him to articulate a word. His vocabulary, however, already extends a long way beyond 'Bow-wow.' To begin with, there are as many different meanings to 'Bow-wow,' or to 'wow' (short and sharp) alone, as some one said a lady could give to the word 'dear,' according to its position in a sentence and the emphasis with which it was pronounced. But, beside saying 'Bow-wow,' the dog whines. And there are many different meanings (which, however, we are sometimes too stupid to understand) in the whining of a dog. We have no fear that dogs or any other of the brute species will furnish competitors for the prizes to be attained by human knowledge; for we observe a barrier between man and brute fixed, and intentionally fixed, by creative power. When we find in the lower creation, as among birds, the power of articulation, there the intelligence is absent which could employ that power for its own development; and where, as in dogs, we find conspicuous tokens of intelligence, there the power of articulation is totally absent. Parrots can be taught to repeat any words, but they never can make up for themselves a new phrase out of the materials in the shape of words that they may have acquired. The natural utterance of many birds, though conveying no meaning to themselves, is distinctly articulate, and sometimes is identical in sound with words that have a meaning to us. But it is the nightingale that possesses the power of articulation to the fullest extent among the species below us. There are races of men whose languages do not employ so many sounds as there are in the nightingale's song. Vowels, consonants of various kinds, sibilants included, even double consonants, as X, Z, are recognized in it by the human ear."

